# The Potential In Vitro Inhibitory Effects of Neurokinin-1 Receptor (NK-1R) Antagonist, Aprepitant, in Osteosarcoma Cell Migration and Metastasis

**DOI:** 10.1155/2022/8082608

**Published:** 2022-09-20

**Authors:** Mohammed Ali Alsaeed, Safieh Ebrahimi, Abbas Alalikhan, Seyedeh Fatemeh Hashemi, Seyed Isaac Hashemy

**Affiliations:** ^1^Department of Clinical Biochemistry, Faculty of Medicine, Mashhad University of Medical Sciences, Mashhad, Iran; ^2^Student Research Committee, Mashhad University of Medical Sciences, Mashhad, Iran; ^3^Surgical Oncology Research Center, Mashhad University of Medical Sciences, Mashhad, Iran

## Abstract

**Background:**

Osteosarcoma, the most frequent osteogenic malignancy, has become a serious public health challenge due to its high morbidity rates and metastatic potential. Recently, the neurokinin-1 receptor (NK-1R) is proved to be a promising target in cancer therapy. This study is aimed at determining the effect of aprepitant, a safe and Food and Drug Administration (FDA) approved NK-1R antagonist, on osteosarcoma cell migration and metastasis, and to explore its underlying mechanism of action.

**Methods:**

Colorimetric MTT assay was employed to assess cell viability and cytotoxicity. A wound-healing assay was used to examine migration ability. The desired genes' protein and mRNA expression levels were measured by western blot assay and quantitative real-time PCR (qRT-PCR), respectively. Gelatinase activity was also measured by zymography.

**Results:**

We found that aprepitant inhibited MG-63 osteosarcoma cell viability in a dose-dependent manner. We also observed that aprepitant inhibited the migrative phenotype of osteosarcoma cells and reduced the expression levels and activities of matrix metalloproteinases (MMP-2 and MMP-9). Aprepitant also reduced the expression of an angiogenic factor, VEGF protein, and NF-*κ*B as an important transcriptional regulator of metastasis-related genes.

**Conclusion:**

Collectively, our observations indicate that aprepitant modulates the metastatic behavior of human osteosarcoma cells, which may be applied to an effective therapeutic approach for patients with metastatic osteosarcoma.

## 1. Introduction

Osteosarcoma is the most frequent osteogenic malignancy, with an annual incidence rate of 4 per million people and high morbidity rates primarily among children and adolescents [1–3]. Osteosarcoma arises from germinal mesenchymal bone-forming cells generating osteoid or immature bone [2, 4]. It is characterized by high metastatic potential and rapid disease progression leading to poor survival outcomes (less than 20%) [5, 6]. Multiple strategies such as surgery, radiation therapy, and adjuvant chemotherapy are recommended as the standard treatments for osteosarcoma patients [5–7]. Despite remarkable advances in these therapeutic approaches, the 5-year survival rate of patients has not improved, partly due to the treatment challenges associated with cancer invasion and metastasis, which imply the need for new effective therapeutic approaches to reduce the metastatic potential of the tumor.

Substance P (SP), a proinflammatory neuropeptide, belongs to the family of tachykinins, and its preferential receptor, neurokinin 1 receptor (NK-1R), have been implicated in the pathogenesis of a wide variety of malignancies [8, 9]. SP regulates tumor cell proliferation, apoptosis, angiogenesis, invasion, and metastasis acting through the NK-1R [9–13]. NK-1R is expressed in a variety of tumor cell lines and tissue samples as well as osteosarcoma cell lines [14, 15]. NK-1R is involved in the viability of tumor cells, and high levels of NK-1R in tumor tissue usually correlate with more aggressive behavior and poor prognosis in many types of human malignancies [9, 16]. Thus, NK-1R is an important mediator of tumorigenesis and represents an interesting anticancer treatment target. Importantly, NK-1R is proved to be a promising target in the suppression of tumor cell invasion and metastasis. Accordingly, different peptide-based and nonpeptide NK-1R antagonists have been developed, and many have revealed potential antitumor effects [17, 18].

Among NK-1R antagonists, aprepitant, a nonpeptide form of NK1R antagonists, is the only one that is approved by the Food and Drug Administration (FDA) for controlling chemotherapy-induced nausea and vomiting (CINV) [19, 20]. We and others previously reported that aprepitant exerts a wide variety of antitumor effects in many human malignancies [20–23]. It has been evidenced that the SP/NK1R system may play an important role in cancer cell invasion and migration in various tumor types and that aprepitant effectively reverses these effects [22, 23]. However, nothing is known about the inhibitory role of aprepitant in osteosarcoma cell invasion and metastasis. Osteosarcoma tumorigenesis is often associated with tumor cell invasion and metastasis, leading to poor prognosis [24, 25]. Therefore, it is interesting to explore the role of aprepitant in osteosarcoma cell invasion and metastasis since it could represent a promising therapeutic strategy and improve the current therapies against osteosarcoma. To this end, this study aims to determine the in vitro anti-metastatic effects of aprepitant and its underlying mechanisms in the MG-63 osteosarcoma cell line.

## 2. Material and Methods

### 2.1. Cell Culture

MG-63 osteosarcoma cell line was acquired from the National Cell Bank of Institute Pasteur of Iran. The cell line was cultured according to the supplier's recommendation, in Dulbecco's Modified Eagle Medium (DMEM), supplemented with 10% heat-inactivated fetal bovine serum (FBS) (Gibco) and 1% antibiotics (penicillin/streptomycin) (Gibco) and incubated in a humidified 37°C incubator with 5% CO_2_. DMEM media was substituted with fresh medium every 3–4 days. For subculturing, cells were harvested by Trypsin/EDTA(Gibco) treatment.

### 2.2. Drugs

Aprepitant was purchased from Sigma–Aldrich (MO, USA) and dissolved in dimethyl sulfoxide (DMSO). SP was also purchased from Sigma–Aldrich Company and dissolved in distilled deionized water, containing 1% bovine serum albumin (BSA). The pH of the mixture was adjusted with 0.05 Molar acetic acid.

### 2.3. MTT Assay

MTT assay was performed to assess aprepitant-mediated cellular cytotoxicity in MG-63 cells. MG-63 cells were seeded at 4 × 10^3^ cells/well and cultured in 96-well plates. After 24 h seeding, aprepitant was added to each well at different concentrations. After 24 h incubation, 20 *μ*l MTT Solution (5 mg/mL) was added to each well, and the plate was incubated for 3 hours at 37°C in the dark. To solubilize the MTT-formazan crystals, the medium was discarded, and 100 *μ*L of DMSO was added to each well and the plate was then incubated at 37°C for 10 minutes. Sample absorbance was then measured at 570 nm using a spectrophotometer ELISA plate reader.

### 2.4. Total RNA Extraction and Quantitative Real-Time Polymerase Chain Reaction (qRT-PCR)

The FavorPrep Blood/Cultured Cell Total RNA Extraction Minikit (YektaTajhiz, Iran) was used to extract total RNA from MG-63 cells. The extracted RNA was then reversely transcribed to complementary DNA (cDNA) using Pars Tous cDNA Synthesis Kit (Iran), following the protocol provided by the manufacturer. qRT-PCR amplification of target genes was done in Roche real-time thermal cycler (Mannheim, Germany) using the related primers and SYBR Green qPCR Master Mix SYBR Green qPCR Master Mix (No ROX). To normalize the expression of the desired genes, a housekeeping gene, Glyceraldehyde-3-phosphate dehydrogenase (GAPDH) was used as an internal reference gene. The 2^−*ΔΔ*Ct^ method was used to analyze the relative changes in gene expression.

### 2.5. Western Blotting

The whole cellular proteins were extracted from MG-63 cells using radioimmunoprecipitation assay (RIPA) buffer, and the protein content was measured by bicinchoninic acid (BCA) spectrophotometric assay. Sodium dodecyl sulfate-polyacrylamide gel electrophoresis (SDS-PAGE) was performed to separate the protein mixture. After protein separation, proteins were immobilized on nitrocellulose (Amersham) membranes. The membranes were then blocked with a 5% blocking solution consisting of nonfat dry milk in Tris-buffered saline (TBS) for 2 h before primary monoclonal antibodies were incubated at 4°C overnight. Afterward, the membranes were exposed to secondary antibodies conjugated with horseradish peroxide for 2 h at room temperature. Finally, the enhanced chemiluminescence (ECL) detection kit (Thermo Fisher Scientific, USA) was employed to reveal protein bands. The protein bands were then quantified by Image J densitometry software.

### 2.6. Wound-healing Assay

MG-63 cell migratory capacity in response to aprepitant was evaluated by wound-healing assay. In this procedure, MG-63 cells were seeded on 6-well plates and incubated until they reached about 90-95% confluence. After getting appropriate confluency, a sterile pipet tip was used to create a scratch wound in the central area of each well. Subsequently, cells were washed twice with PBS and then treated with the desired aprepitant concentration. Finally, generated wounds were photographed at 0, 24, and 48 h, and the images were then analyzed and plotted by Image J software.

### 2.7. Zymography

Gelatin-zymography was carried out to detect the effects of aprepitant on MMPs activity in MG-63 cells. 10% polyacrylamide gel electrophoresis containing gelatin (1 mg/mL) was prepared and loaded with 24 h-cultured supernatants. Following SDS-PAGE, the gels were washed gently for 1 hour with 2.5% Triton X-100 to remove SDS. The gels were then incubated overnight in 50 ml incubation buffer of (% Triton X-100, 50 mM Tris HCl pH 7.5,5 mM CaCl2,1 *μ*M ZnCl2) at 37°C. After 24 h incubation, gels were stained in Coomassie Brilliant Blue (R250) for 1 h, and subsequently destained with 40% methanol and 10% acetic acid until white bands, indicating the degradative activity of MMPs, appeared. Finally, gels were scanned by the Gel-Doc system, and a densitometric assessment of bands was performed using the ImageJ software.

### 2.8. Statistical Analysis

Statistical assessment was performed using GraphPad Prism 8. Statistical tests (independent-samples *t*-test or one-way ANOVA) were used to analyze the statistical differences. All results were evinced as mean ± standard deviation (SD). The *P* value less than 0.05 (*P* < 0.05) was regarded statistically significant.

## 3. Results

### 3.1. Aprepitant Efficiently Suppressed the Proliferation of MG-63 Osteosarcoma Cell Line

**Figure 1:**
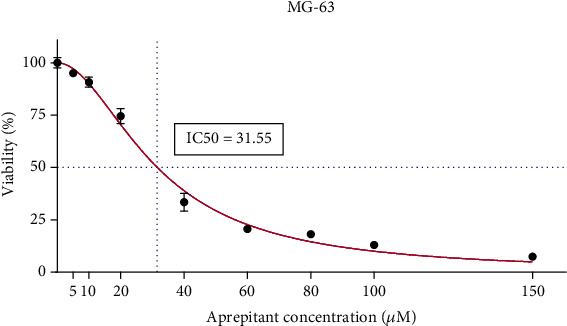
The effects of aprepitant on the MG-63 osteosarcoma cell viability.

We first determined the effect of aprepitant on the cell viability of cultured MG-63 cells, using the MTT assay. MG-63 cells were exposed to various doses of aprepitant (5, 10, 20, 40, 60, 80, 100, and 150 *μ*M) and incubated for 24 h. As shown in Figure 1, aprepitant treatment significantly reduced the viability of osteosarcoma cells in a dose-dependent manner. The concentration of 31.55 *μ*M inhibited the growth of MG-63 cells by 50% after 24 h. Accordingly, the concentrations of aprepitant which cause about 50% of growth inhibition (IC50), and half of the IC50, were selected for the following experimental approaches. Our intention was to test the effects of aprepitant at both sublethal (1/2 IC50) and lethal (IC50) concentrations.

Cultured MG-63 cells were exposed to various doses of aprepitant (ranging from 5 to 150 *μ*M) for 24 h, and cell viability was evaluated by MTT assay. The results indicate that aprepitant treatment significantly reduced the viability of osteosarcoma cells in a dose-dependent manner. The data shown here represent three independent experiments that yielded similar results.

### 3.2. Aprepitant Efficiently Suppressed the Migratory Phenotype of MG-63 Osteosarcoma Cell Line

**Figure 2:**
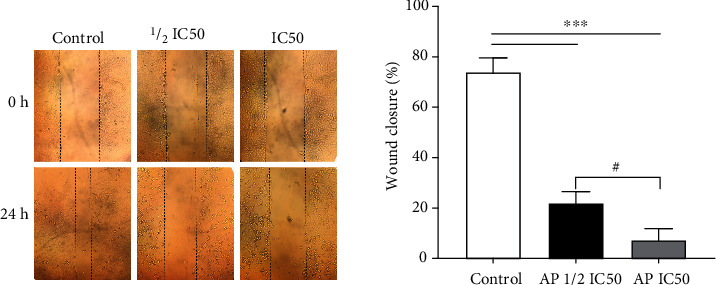
The effect of aprepitant on MG-63 cells migration. Migration ability was examined by wound-healing assay. The results showed that after 24 h, aprepitant-treated cells at the concentration of IC50 and 1/2 IC50 had larger wound distances than that of the control group. The percentage of wound closure was analyzed using the Image J software and plotted. (^∗^*P* ≤ 0.05; ^∗∗^*P* ≤ 0.01; ^∗∗∗^*P* ≤ 0.001; ^∗∗∗∗^*P* ≤ 0.0001) indicated statistical significance compared to the control group. (#*P* ≤ 0.05 indicating statistical significance between two aprepitant-treated groups (IC50 and 1/2 IC50).

Osteosarcoma tumorigenesis is often associated with tumor cell migration leading to metastasis and further decreased patient survival [24, 25]. Therefore, in vitro wounding healing assay was used to determine the capability of MG-63 cells migration in response to aprepitant. The wound-healing assay results indicated that after 24 h of treatment, aprepitant dose-dependently reduced the extent of wound closure (Figure 2(a)), suggesting that aprepitant could inhibit the migration of osteosarcoma cells.

### 3.3. Aprepitant Efficiently Inhibited the Expression Levels and Activities of Matrix Metalloproteinases (MMPs) in MG-63 Osteosarcoma Cell Line

**Figure 3:**
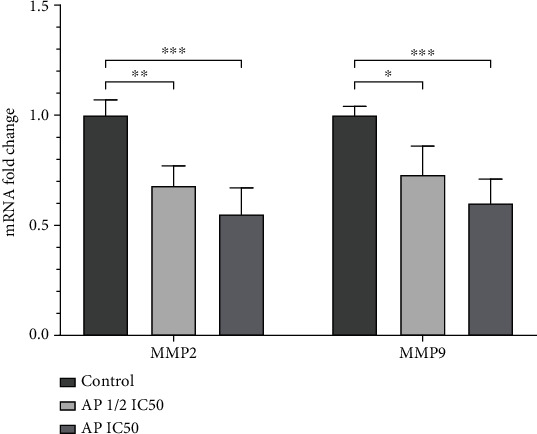
Aprepitant inhibits the expression of MMP-2 and MMP-9 in the MG-63 osteosarcoma cell line. MG-63 cells were treated with aprepitant (AP) at the concentration of IC50 and 1/2 IC50 for 24 h, and subsequently, the mRNA expression levels of MMP-2 and MMP-9 were evaluated by qRT-PCR. The experiments were conducted in triplicates, and the results are illustrated as the mean ± SD. (^∗^*P* ≤ 0.05; ^∗∗^*P* ≤ 0.01; ^∗∗∗^*P* ≤ 0.001; ^∗∗∗∗^ *P* ≤ 0.0001), indicating statistical significance compared to the control group.

The matrix metalloproteinases (MMPs) including MMP-2 and MMP-9 belong to a family of proteinases that can catalyze the cleavage of extracellular matrix components, thus favoring tumor cell migration, invasion, and metastasis [26–28]. To further investigate the antimigrative effects of aprepitant, we evaluated the mRNA expression levels of matrix metalloproteinases (MMPs), MMP-2, and MMP-9, in MG-63 cells in response to different concentrations of aprepitant by qRT-PCR. As illustrated in Figure 3, aprepitant significantly reduced the expression levels of MMP-2 and MMP-9 in a dose-dependent manner.

**Figure 4:**
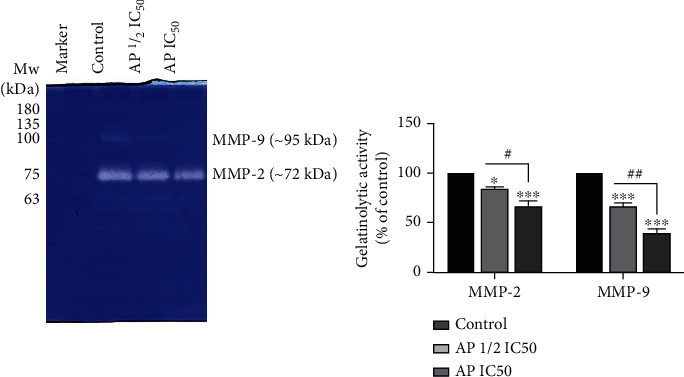
Analysis of MMP-2 and MMP-9 activity in osteosarcoma cells in response to different aprepitant(AP) concentrations. Gelatinase activity of MMP-2 and MMP-9 were revealed as transparent bands against a blue background in the MG-63 osteosarcoma cell line. Zymographic bands were analyzed by computer ImageJ software and plotted. (^∗^*P* ≤ 0.05; ^∗∗^*P* ≤ 0.01; ^∗∗∗^*P* ≤ 0.001; ^∗∗∗∗^*P* ≤ 0.0001) indicated statistical significance compared to the control group.

We also examined whether aprepitant could influence the gelatinase activities of MMP-2 and MMP-9 in the MG-63 osteosarcoma cell line. MG-63 cells were treated with aprepitant (AP) at the concentration of IC50 and 1/2 IC50 for 24 h, and gelatinase activity was assessed by zymography. As illustrated in Figure 4, aprepitant significantly reduced the enzymatic activity of MMP-2 and MMP-9 in a dose-dependent manner. Altogether, these results suggest that aprepitant can modulate the invasive behavior of human osteosarcoma cells, possibly by regulating both the expression and activities of MMP-9 and MMP-2.

### 3.4. Aprepitant Efficiently Inhibited the Expression Level of Vascular Endothelial Growth Factor-a (VEGF-A) in MG-63 Osteosarcoma Cell Line

**Figure 5:**
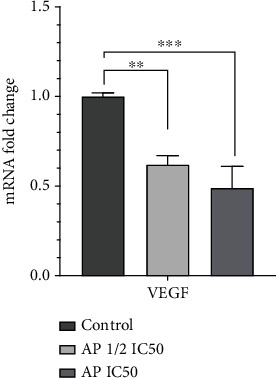
Aprepitant inhibits the expression of VEGF-A in the MG-63 osteosarcoma cell line. MG-63 cells were treated with aprepitant (AP) at the concentration of IC50 and 1/2 IC50 for 24 h, and subsequently, the mRNA expression level of VEGF-A was evaluated by qRT-PCR. The experiments were conducted in triplicates, and the results are illustrated as the mean ± SD. (^∗^*P* ≤ 0.05; ^∗∗^*P* ≤ 0.01; ^∗∗∗^*P* ≤ 0.001; ^∗∗∗∗^*P* ≤ 0.0001) indicated statistical significance compared to the control group.

**Figure 6:**
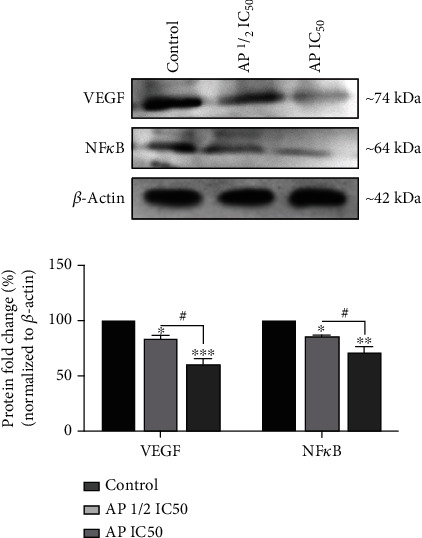
Aprepitant reduces the protein expression of VEGF and NF-*κ*B. MG-63 cells were treated with aprepitant (AP) at the concentration of IC50 and 1/2 IC50 for 24 h, and subsequently, the protein expression levels of c VEGF and NF-*κ*B were assessed by western blotting. Western blot was conducted at least twice, and a representative picture is indicated. Western blot bands were quantitated by computer ImageJ software, plotted, and compared. (^∗^*P* ≤ 0.05; ^∗∗^*P* ≤ 0.01; ^∗∗∗^*P* ≤ 0.001; ^∗∗∗∗^*P* ≤ 0.0001) indicated statistical significance compared to the control group.

Osteosarcoma tumor growth and metastasis are closely associated with angiogenesis [29]. Furthermore, MMPs have been shown to support tumor angiogenesis by releasing proangiogenic factors like VEGF-A [28]. Consequently, we investigated whether aprepitant can also affect the expression of the VEGF-A in osteosarcoma cells. Our results indicated that aprepitant reduced the expression of mRNA (Figure 5) and protein (Figure 6) levels of the VEGF-A in MG-63 osteosarcoma cells in a dose-dependent manner. These observations suggest that aprepitant could affect the process of angiogenesis and can be developed as an antiangiogenic agent.

### 3.5. Aprepitant Efficiently Inhibited the Protein Level of Nuclear Factor Kappa B (NF-*κ*B) in MG-63 Osteosarcoma Cell Line

NF-*κ*B is a crucial transcription factor associated with cancer cell invasion and metastasis. Furthermore, NF-*κ*B has been reported as an upstream target stimulating the expression of MMP-2, MMP-9, and VEGF [30, 31]. We hypothesized that the aprepitant's antimetastatic effects might be mediated by the inhibition of NF-*κ*B. To examine this possibility, MG-63 cells were treated with aprepitant (AP) at the concentration of IC50 and 1/2 IC50 for 24 h, and subsequently, the protein expression levels of NF-*κ*B were assessed by western blotting. As indicated in Figure 6, aprepitant significantly reduced the protein expression of NF-*κ*B in MG-63 osteosarcoma cells in a dose-dependent manner. Thus, the inhibition of NF-*κ*B as a key transcriptional regulator involved in tumor angiogenesis and metastasis could be one of the antimetastatic mechanisms of aprepitant.

## 4. Discussion

In the present study, we examined the potential in vitro inhibitory effects of aprepitant in osteosarcoma cell invasion and metastasis. We found several lines of evidence suggesting that aprepitant may be considered as an antimetastatic agent in osteosarcoma. First, aprepitant efficiently suppresses the migratory phenotype of cultured osteosarcoma cells. Second, aprepitant efficiently inhibits the expression levels and activities of MMP-2, and MMP-9, which are known as specific metastasis-promoting factors. Third, aprepitant suppresses the expression of an angiogenic factor, VEGF-A protein, which is known to promote tumor angiogenesis and migration. Finally, aprepitant suppresses the expression of NF-*κ*B as an important transcriptional regulator of MMPs and angiogenic factors.

Cancer cell migration and metastasis are among the main causes of osteosarcoma patients' poor prognosis [24, 25]. Thus, there is a need for new effective therapeutic approaches targeting the process of metastasis. Recently, the neurokinin-1 receptor (NK-1R) is proved to be a promising target in cancer therapy and in particular regulation of cancer invasion and metastasis. Accordingly, different peptide-based and nonpeptide NK-1R antagonists have been developed, and many have revealed potential antimetastatic effects [19, 20]. In this line, Ma et al. indicated that the NK-1R antagonist, L-733,060, inhibited SP-induced expression of VEGF-C and MMP-9 in endometrial adenocarcinoma cells, thus reducing tumor invasion and metastasis [32]. Consistently, NK-1R antagonists, L-733,060 and L-732,138, could also inhibit SP-induced expression of MMP-2 in pancreatic cancer cells and thus impairing cancer migration [33].

NK-1R antagonists have been also reported to inhibit the metastasis-promoting genes, including MMPs, and EMT-inducing genes in head and neck cancers [34]. NK-1R antagonist, L703606, could also attenuate the expression of metastasis-related factors like MMP9 in gallbladder cancer cells [35]. These data combined highlight the therapeutic potential of targeting NK-1R in controlling tumor invasion and metastasis.

Among NK-1R antagonists, aprepitant, a nonpeptide form of NK1R antagonists, is only approved by the Food and Drug Administration (FDA) for controlling chemotherapy-induced nausea and vomiting (CINV) [20, 36]. Previous in vitro observations described the antimetastatic effects of aprepitant [20]. Aprepitant has led to considerable therapeutic effects in melanoma by inhibiting tumor cell growth, angiogenesis, and tumor cells invasion and metastasis [37]. Aprepitant also suppresses cell motility and invasion by affecting membrane blebbing [38]. Membrane blebbing is an important process in cell movement, in tumor cell invasion, and is mediated through the Rho-activated ROCK system [39]. It should be noted that application of the aprepitant was reported to be safe and without any adverse toxicological effects at all tested doses even at excessively large doses [36, 40]. Therefore, there is a great interest in identifying the therapeutic value of aprepitant in osteosarcoma.

We, therefore, examined the therapeutic potential of targeting NK-1R in controlling osteosarcoma invasion and metastasis by aprepitant. Muñoz et al. have previously indicated that NK-1R is significantly expressed both at the protein and mRNA in MG-63 osteosarcoma cell line. That NK-1R blockade with different NK1R antagonists as well as aprepitant exerted strong growth inhibitory effects both in vitro and in vivo [15]. Consistently, we showed similar growth inhibitory capacity of aprepitant on MG-63 cells in vitro. Since osteosarcoma treatment challenges are mainly associated with cancer invasion and metastasis, we examined the metastasis inhibitory capacity of aprepitant on MG-63 cells in vitro. We observed that aprepitant inhibited the migrative phenotype of osteosarcoma cells and reduced the expression levels and activities of matrix metalloproteinases (MMP-2 and MMP-9). In accordance, we previously indicated that aprepitant reduces the migratory phenotype of different tumor types including esophageal squamous cell carcinoma [41], cervical cancer, [23], and prostate cancer [22] which was correlated with reduced expression of MMP-2 and MMP-9. MMPs are closely related to tumor invasion and metastasis, and are necessary for tumor cells to migrate and invade surrounding tissues, ultimately establishing distant metastases [27, 28]. Furthermore, MMP-2 and MMP-9 have been found as potential therapeutic targets in different tumor types as well as osteosarcoma [27]. Therefore, our observations in this context strengthen the antimetastatic role of aprepitant in osteosarcoma through the regulation of MMPs.

Angiogenesis plays a critical role in osteosarcoma progression and metastasis. VEGF is the main angiogenic factor in tumor angiogenesis, supporting tumor growth and metastasis [29]. It has been shown that VEGF and MMP-9 expression in osteolytic lesions of bone is associated with the higher risk of local recurrence and bone destruction [42]. We, therefore, examined the antiangiogenetic effect of aprepitant and found reduced expression of VEGF-A in MG-63 cells treated with aprepitant. In agreement with our results, modulation of VEGF-A and its receptor expression by aprepitant has been previously documented [41]. Furthermore, therapeutic targeting with aprepitant led to inhibition of in vivo angiogenesis and subsequent metastasis in hepatoblastoma [43]. It can therefore be concluded that inhibition of angiogenesis is one mechanism underlying the antimetastatic effect of aprepitant. Since angiogenesis has a critical role in osteosarcoma tumor progression, finding new antiangiogenic drugs in the management of osteosarcoma is required. Tyrosine kinase inhibitor, lenvatinib, alone or in combination with other agents has been found as a potent anti-VEGFR agent in the primitive bone tumor, including giant cell tumors of bone (GCTB) [44]. Moreover, the efficacy of lenvatinib in combination with pembrolizumab or ifosfamide/etoposide is currently under evaluation in patients with refractory or relapsed osteosarcoma [45]. Considering the potent anti-VEGF activity of aprepitant, a combination of lenvatinib with aprepitant may also have a synergistic effect in osteosarcoma. Thus, it would be interesting to evaluate the combination of lenvatinib with aprepitant in future studies.

NF-*κ*B signaling plays an important role in regulating several biological activities that may affect several steps in osteosarcoma metastasis [46, 47]. Importantly, NF-*κ*B has been suggested as a direct transcriptional regulator of MMPs and angiogenic factors [30, 31, 48]. We explored next whether aprepitant can affect the expression of NF-*κ*B. We found that aprepitant significantly reduced the protein expression of NF-*κ*B in MG-63 cells. It is, therefore, possible that aprepitant can regulate NF-*κ*B and further lead to changes in the NF-*κ*B downstream targets, including MMP-2, MMP-9, and VEGF-A. However, further in-depth investigations are required to verify the potential linkage of aprepitant to this pathway and associated metastasis.

## 5. Conclusion

**Figure 7:**
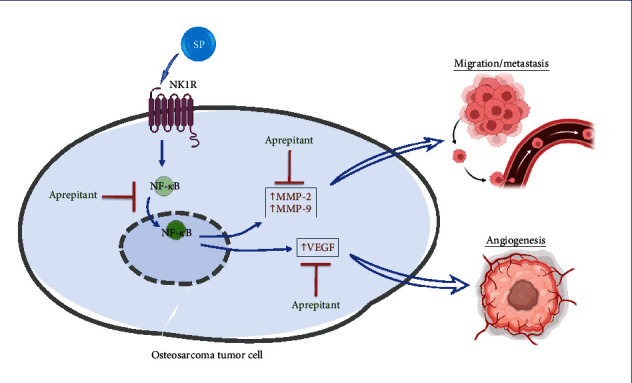
This graphical abstract illustrates the possible mechanisms through which neurokinin-1 receptor (NK-1R) antagonist, aprepitant, exerts its anti-tumoral effects in osteosarcoma cell.

Here, we found that aprepitant leads to considerable in vitro therapeutic effects in osteosarcoma by inhibiting tumor cell growth, angiogenesis, migration, and metastasis (Figure 7). We suggested that the antimetastatic effect of aprepitant in osteosarcoma is probably attributed to its ability to modulate the transcriptional regulator, NF-*κ*B, and subsequently, its target genes, including MMP-2, MMP-9, and VEGF-A. However, more broadly research and further in vivo work are required to establish this. Given that aprepitant has a good safety profile against normal cells [23, 43], following with further studies to identify its anticancer effects would be worthwhile for future of osteosarcoma therapy and research.

Thus, it is encouraging to explore the following issues for future research: First, application of the aprepitant for chemoprevention of metastasis development. Having significant anti-metastatic capabilities, aprepitant combined with other chemotherapeutic drugs may also bring potential benefits against osteosarcoma. Future studies on this issue are therefore recommended. Second, various signaling pathways and transcriptional factors are significantly altered in the many cellular processes of migration, invasion, and metastasis in osteosarcoma. However, we only explored the function of NF-*κ*B and its target genes. Further studies with more focus on other metastasis-promoting factors are therefore suggested. Finally, it is suggested that the aprepitant mediated- therapeutic effects are verified in future in vivo studies.

## Data Availability

Data are available upon request.
